# The Dual Roles of Protein-Bound Solutes as Toxins and Signaling Molecules in Uremia

**DOI:** 10.3390/toxins14060402

**Published:** 2022-06-11

**Authors:** Rosalinde Masereeuw

**Affiliations:** Division of Pharmacology, Utrecht Institute for Pharmaceutical Sciences, Utrecht University, 3584 CG Utrecht, The Netherlands; r.masereeuw@uu.nl

**Keywords:** protein-bound uremic toxins, gut-kidney axis, renal secretion, organic anion transport, remote signaling

## Abstract

In patients with severe kidney disease, renal clearance is compromised, resulting in the accumulation of a plethora of endogenous waste molecules that cannot be removed by current dialysis techniques, the most often applied treatment. These uremic retention solutes, also named uremic toxins, are a heterogeneous group of organic compounds of which many are too large to be filtered and/or are protein-bound. Their renal excretion depends largely on renal tubular secretion, by which the binding is shifted towards the free fraction that can be eliminated. To facilitate this process, kidney proximal tubule cells are equipped with a range of transport proteins that cooperate in cellular uptake and urinary excretion. In recent years, innovations in dialysis techniques to advance uremic toxin removal, as well as treatments with drugs and/or dietary supplements that limit uremic toxin production, have provided some clinical improvements or are still in progress. This review gives an overview of these developments. Furthermore, the role protein-bound uremic toxins play in inter-organ communication, in particular between the gut (the side where toxins are produced) and the kidney (the side of their removal), is discussed.

## 1. Introduction

Over the years, research in the field of nephrology led to advances in the treatment of patients suffering from end-stage kidney disease (ESKD) who rely on dialysis as renal replacement therapy, but the mortality rate after two years remains close to 50% and significant steps that also impact their quality of life have, as of yet, to be taken. The need for improved treatment options becomes even more urgent with the global rise in the number of patients, as it has been estimated that by 2030 approximately 5 million people are expected to depend on renal replacement therapies [1,2]. This, consequently, places a burden on health care systems and society. Hemodialysis was first introduced by Willem Kolff in 1945 who developed a machine consisting of a rotating drum kidney in a static open bath with 20 m of cellophane dialysis tubing wrapped around it. In the following 75 years, the therapy has been brought into daily clinical practice through innovations in the semi-permeable membrane of the dialyzer. Other technological improvements have included the safety of the dialysis monitors, the use of convective techniques such as hemodiafiltration, ultrapure water, and the introduction of bicarbonate as a buffer. Dialysis treatment has improved the survival of patients with kidney failure but has had limited effects on their well-being. In healthy kidneys, protein-bound solutes are *secreted* by the kidney’s proximal tubule cells and can be completely cleared from the initial filtrate, a phenomenon already described mid-20th century by Homer Smith [3]. Hence, the current dialysis therapy is limited in that only the free fraction of the albumin-bound uremic toxins are removed and the bound fractions, for some toxins over 90%, are retained, as albumin is too large to pass through the pores of the dialyzers.

## 2. Dialysis Technology Advancements to Enhance Toxin Removal

To loosen the binding of uremic toxins, several innovative developments have been explored, including electrically triggered dissociation of protein-toxin complexes with incorporated carbon nanotubes in a hemodialysis device system [4]. Such a device could provide an improved toxin removal with low protein loss, but it is still in development. Moreover, experimentally enhancing the ionic strength of plasma can efficiently release uremic wastes from protein binding, making them available for dialyzer removal, but this approach bears the risk of plasma sodium retention [5]. Another approach has examined the effect of enhancing the efficiency of hemodialysis by the infusion of a competitor that competes with the toxins for the albumin binding site(s) into the arterial blood line, increasing the toxins’ free fraction. This promising approach was recently tested using ibuprofen as a competitor, leading to reduced serum levels of indoxyl sulfate and p-cresol sulfate through their increased dialytic removal [6]. Further, it is known that the binding of many solutes to albumin is pH-dependent. Accordingly, it has been reported that preventing the increased pH of the plasma that regularly occurs during standard hemodialysis (from 7.4 to 7.49) might increase the dialytic removal of kynurenine [7]. Further reduction in dialysate pH would be expected to enhance the dialytic removal of a number of other protein-bound solutes.

Uremic toxin removal could be enhanced if dialysis was more prolonged and employed by membranes with greater porosity, which is the aim of portable and wearable systems. This might significantly improve outcomes and quality of life for ESKD patients. Such devices will be beneficial for application in hemodialysis as well as peritoneal dialysis but require a dialysate regenerating system and highly efficient membranes to clear the uremic toxins. Still, regeneration of spent dialysate is challenging considering the accumulating ions, urea, and organic solutes in a relatively small dialysate volume, for which purification strategies include enzymatic hydrolysis of urea by urease, electro-oxidation, and sorbent techniques [8]. Moreover, membranes should be ultrathin to guarantee a large surface area in a small device and should have sufficient strength and appropriate pore sizes to allow middle molecular weight molecules’ removal. This might be offered by nanoporous silicon-nitride-based membranes, with a diameter of pores typically 100 nanometers or smaller [9]. Novel membranes that contain mesoporous carbons, i.e., nanoporous material containing pores with diameters between 2 and 50 nm, as sorbents with dual-porosity (micro/meso) or sorbent particles for selective and efficient removal of protein-bound uremic toxins as well as cytokines from human plasma, may provide promising alternatives [10,11,12].

## 3. Interventions to Reduce Plasma Levels of Uremic Toxins

Several uremic toxins are the product of gut bacterial digestion of tryptophan and tyrosine to non-polar metabolites that enter the hepatic portal circulation and are sulfated and hydrogenated to yield the postulated uremic solutes, indoxyl sulfate, p-cresyl sulfate, and kynurenine. Charcoal, in the form of the carbonaceous adsorbent, AST-120, in the bowel serves to trap indole, the precursor of these uremic toxins preventing their absorption. In early studies, the oral intake of AST-120 seemed promising in reducing the plasma concentration of the protein-bound uremic toxin, indoxyl sulfate, but effects on other clinical endpoints, such as slowing disease progression and all-cause mortality, were less clearcut [13]. Clearly, the treatment of ESKD is much more complex than the reduction in the plasma level of a single uremic toxin. Potentially, the use of antibiotics to suppress the gut microbiota might also reduce the plasma levels of uremic toxins [14]. In a recent study, Nazzal and coworkers found that the small weekly dose of vancomycin (250 mg/week) resulted in a reduction in the plasma concentration of 7 colon-derived solutes. They described a significant effect of vancomycin on the gut microbiome structure with a decrease in alpha diversity and a change in beta diversity. Multiple taxa decreased with vancomycin, including genera Clostridium and Bacteroides. This observation awaits further investigation into the clinical outcome. Furthermore, a recent systematic review revealed that the treatment of dialysis patients with probiotics, prebiotics, or synbiotics may lead to reduced uremic toxin plasma levels and alleviated inflammation [15].

## 4. Biohybrid Solutions

**Figure 1:**
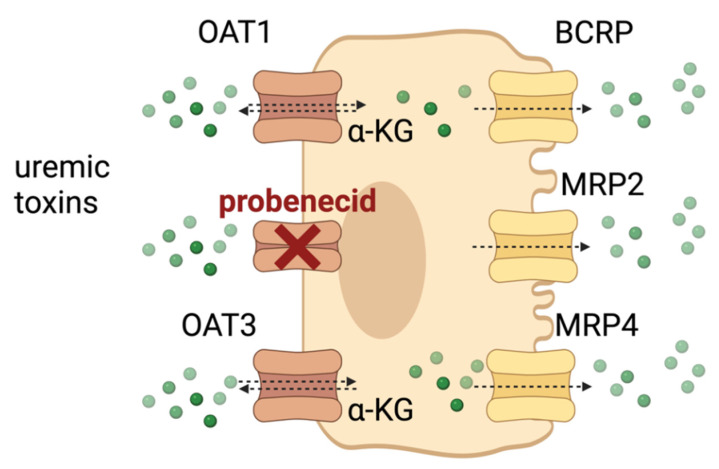
Schematic illustration of the proximal tubule epithelial cell as the site for active uremic toxin secretion. Organic Anion Transporters 1 and 3 (OAT1/3) transport a broad range of anionic uremic toxins into the cell in exchange for α-ketoglutarate (α-KG), while the efflux pumps Breast Cancer Resistance Protein (BCRP) and the Multi-Drug Resistance-associated Proteins 2 (MRP2/4) are responsible for excretion into the tubular lumen for final removal. Competition with inhibitors (such as probenecid, or co-administered drugs) may compromise tubular secretion. Created with BioRender.com (accessed on 10 June 2022).

It has been documented that the reduced renal clearance of protein-bound uremic toxins is associated with kidney disease progression and all-cause mortality, even after adjustment for eGFR, albuminuria, and other confounding characteristics [16]. Therefore, adding an active tubular component to (optimized) filtration/dialysis might lead to an improved outcome for ESKD patients [17,18,19]. These biohybrid approaches (renal assist devices) are encouraging developments, though still not widely available. Kidney proximal tubule cells are equipped with basolateral organic anion transporters (OATs) that selectively take up protein-bound toxins from the blood and actively secrete them into the renal tubular lumen [20,21,22]. In the organic anion transport pathway, organic anion transporters 1 and 3 (OAT1/3) at the basolateral membrane [23], and breast cancer resistance protein (BCRP) and multidrug resistance-associated transporters 2 and 4 (MRP2/4) at the apical membrane [24], have been implicated in this excretion process. Free toxins compete for the OAT’s binding sites, with a higher affinity for the transport proteins compared to albumin favoring the toxin’s cellular uptake [25]. The high-capacity efflux pumps, BCRPs and MRPs, then accomplish the final removal (Figure 1). Making use of this natural process and the (intra) cellular regulatory machinery that drives the transporters involved might offer targets for the removal of protein-bound uremic toxins.

## 5. Remote Sensing and Signaling: Linking the Kidney to the Gut

**Figure 2:**
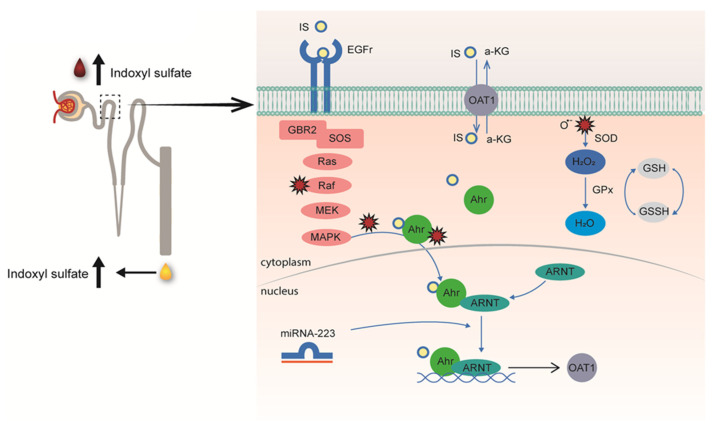
Schematic illustration of the remote metabolite sensing and signaling pathway as an effective OAT1 regulation mechanism to maintain plasma indoxyl sulfate levels by controlling their secretion. Proximal tubule cells in kidneys sense elevated uremic toxin levels through epidermal growth factor (EGF) receptors and down-stream signaling to induce their secretion by upregulating the organic anion transporter 1 (OAT1) via AhR and EGFR signaling, controlled by miR-223. Concomitantly produced reactive oxygen species (ROS) control OAT1 activity, balanced by the glutathione pathway, as confirmed by cellular metabolomic profiling (from [28]).

The transport of solutes is not restricted to the kidneys, as most tissues express multi-specific membrane transporters. These, together with metabolism enzymes, form a multi-scale and adaptive communication network that is important for maintaining homeostasis. The Remote Sensing and Signaling Theory, pioneered by Sanjay Nigam and coworkers, supports the central role of transporters also in the progression of the uremic syndrome in severe kidney failure [26,27]. In healthy kidneys, tubular cells sense elevated plasma levels of uremic toxins and react by increasing transporter expression levels to support toxin excretion, as was demonstrated for indoxyl sulfate [28]. This study was preceded by an observation of human volunteers subjected to a high protein diet for 14 days who showed elevated plasma levels of tryptophan and tyrosine-derived metabolites, in particular indoxyl sulfate, accompanied by an enhanced urinary indoxyl sulfate excretion [29], that could be explained by an increase in renal OAT1 expression [28]. The upregulation of the uremic toxin transporter was the result of the aryl-hydrocarbon receptor (AhR) activation, a ligand-dependent transcription factor that can induce the expression of membrane transporters [30], as well as the epidermal growth factor receptor (EGFR), a receptor previously implicated in OAT1 regulation [31]. The two-hit mechanism by which indoxyl sulfate induces its own secretion involves further a highly sophisticated inter-organ communication pathway. On the one hand, after (extracellular) binding, indoxyl sulfate stimulates EGFR signaling with downstream signaling via the MAPK-ERK pathway and the aryl hydrocarbon nuclear translocator (ARNT), while, at the same time, the uremic solute activates AhR after OAT1-mediated uptake. Subsequently, (intracellularly) activated AhR complexes with ARNT translocate to the nucleus to induce OAT1 expression, a mechanism positively regulated by miR-223 and reactive oxygen species (Figure 2). Interestingly, serum levels of miR-223 were significantly lower in patients with advanced kidney disease [32]. This non-coding RNA is a hematopoietic factor with a regulatory role in cholesterol and cardiac homeostasis and serves as a negative feedback mechanism controlling excessive immune responses. These findings support the inter-organ communication in the complex pathophysiology of the uremic syndrome that might also offer targets for interventions in disease progression.

In CKD, this adaptive response is lost, and a decrease in transporter expression is observed [33], possibly caused by aberrant gut-to-kidney signaling through increased blood concentrations of uremic toxins [34]. This assumption is further supported by recent observations in which protein-bound uremic toxins may compete with co-administered drugs commonly used in CKD management, which potentially compromise the residual tubular function [25,35,36]. The progressive loss of tubular function and accompanied increase in retention of uremic toxins fuel systemic metabolic disorders and comorbidities associated with the pathophysiology of CKD.

**Figure 3:**
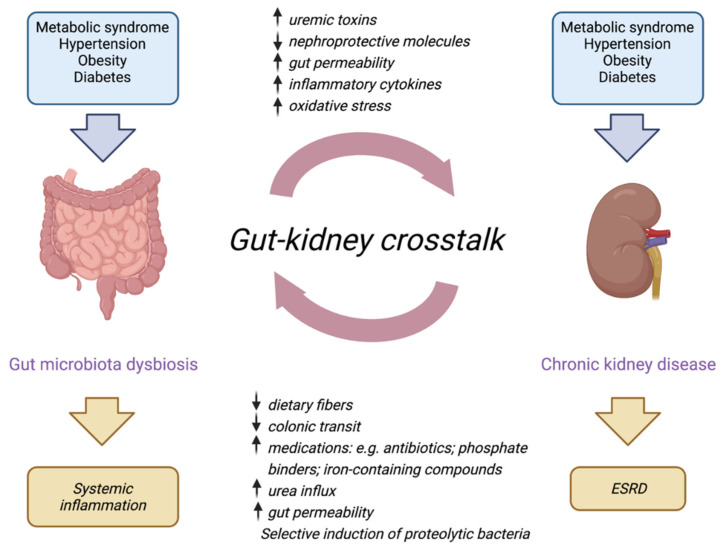
Schematic illustration of the gut–kidney axis in chronic kidney disease. Mechanisms at which gut microbiota and chronic kidney disease communicate through the gastrointestinal–kidney axis. (Created with BioRender.com (accessed on 10 June 2022), Reprinted with permission from Ref. [39]. Copyright 2020 Nature Springer (Pflügers Archiv—European Journal of Physiology, Natalia Lucía Rukavina Mikusic, Nicolás Martín Kouyoumdzian & Marcelo Roberto Choi)).

While targeting uremic toxins clearance in kidney disease has gained much interest, the exact relation between toxins and associated comorbidities, such as cardiovascular complications, is still unclear, and it remains to be determined whether enhancing toxin removal will improve clinical outcomes and patients’ quality of life [37]. Perhaps, we should put more emphasis on reducing the production of uremic toxins in parallel with improving their clearance. The retention solutes originate mostly from endogenous and (colonic) microbial metabolism, and specifically, the bacterial metabolites, phenols, indoles, and amines (i.e., indoxyl sulfate and p-cresyl sulfate) are potentially toxic [38]. Recently, uremia was shown to be associated with an altered gut microbiome composition, as well as altered metabolism, leading to increased production of uremic toxins [39], thereby linking the kidney to the gut (Figure 3). Moreover, the accumulating uremic toxins in plasma may directly affect the intestinal barrier [38], although it is more likely that urease-containing bacteria contribute to the intestinal barrier disruption [40,41]. It is currently unclear how we can influence the gut microbiome and metabolome to achieve a reduced disease burden. Acquiring more information regarding the pathways that can be nutritionally or pharmacologically triggered, for instance using advanced in vitro models [42], will bring us closer to this aim.

## 6. Conclusions

Innovations in the semi-permeable membrane of the dialyzer for renal replacement therapy, the use of hemodiafiltration, ultrapure water, and the introduction of bicarbonate as a buffer have improved the safety of dialysis therapies but not the quality of life for CKD patients. Interventions to reduce plasma levels and protein binding of uremic toxins, including treatment of dialysis patients with probiotics, prebiotics, or synbiotics, or a reduction in dialysate pH may alleviate symptoms by reducing plasma uremic toxin levels or increasing their dialytic removal, but both approaches warrant further investigation. Biohybrid solutions, combining kidney tubular epithelial cells with a dialyzer, may offer another solution in the future. These cells are equipped with membrane transporters and receptors that are responsible for balancing uremic toxin levels in homeostasis. Through remote metabolite sensing and signaling, it appeared that gut microbiome-derived toxins can regulate their renal excretion. However, in kidney failure, the same metabolites are responsible for systemic toxicity. This dual role of uremic toxins should be understood in sufficient detail to allow interventions at preserving kidney tubular function in patients to reduce the progression of kidney disease.

## Data Availability

Not applicable.
